# Shear‐dependent platelet aggregation size

**DOI:** 10.1111/aor.13783

**Published:** 2020-08-15

**Authors:** Chris Hoi Houng Chan, Masataka Inoue, Katrina K. Ki, Tomotaka Murashige, John F. Fraser, Michael J. Simmonds, Geoff D. Tansley, Nobuo Watanabe

**Affiliations:** ^1^ School of Engineering and Built Environment Griffith University Gold Coast QLD Australia; ^2^ Critical Care Research Group The Prince Charles Hospital Brisbane QLD Australia; ^3^ Faculty of Medicine University of Queensland Brisbane QLD Australia; ^4^ Department of Life Sciences Systems Engineering and Science Graduate School of Engineering and Science Shibaura Institute of Technology Saitama Japan; ^5^ School of Engineering Tokyo Institute of Technology Tokyo Japan; ^6^ Menzies Health Institute Queensland Griffith University Gold Coast QLD Australia; ^7^ School of Medicine Griffith University Gold Coast QLD Australia

**Keywords:** ADAMTS13, exposure time, platelet aggregate, shear rate, von Willebrand factor

## Abstract

Nonsurgical bleeding is the most frequent complication of left ventricular assist device (LVAD) support. Supraphysiologic shear rates generated in LVAD causes impaired platelet aggregation, which increases the risk of bleeding. The effect of shear rate on the formation size of platelet aggregates has never been reported experimentally, although platelet aggregation size can be considered to be directly relevant to bleeding complications. Therefore, this study investigated the impact of shear rate and exposure time on the formation size of platelet aggregates, which is vital in predicting bleeding in patients with an LVAD. Human platelet‐poor plasma (containing von Willebrand factor, vWF) and fluorochrome‐labeled platelets were subjected to a range of shear rates (0‐10 000 s^−1^) for 0, 5, 10, and 15 minutes using a custom‐built blood‐shearing device. Formed sizes of platelet aggregates under a range of shear‐controlled environment were visualized and measured using microscopy. The loss of high molecular weight (HMW) vWF multimers was quantified using gel electrophoresis and immunoblotting. An inhibition study was also performed to investigate the reduction in platelet aggregation size and HMW vWF multimers caused by either mechanical shear or enzymatic (a disintegrin and metalloproteinase with a thrombospondin type 1 motif, member 13—ADAMTS13, the von Willebrand factor protease) mechanism under low and high shear conditions (360 and 10 000 s^−1^). We found that the average size of platelet aggregates formed under physiological shear rates of 360‐3000 s^−1^ (200‐300 μm^2^) was significantly larger compared to those sheared at >6000 s^−1^ (50‐100 μm^2^). Furthermore, HMW vWF multimers were reduced with increased shear rates. The inhibition study revealed that the reduction in platelet aggregation size and HWM vWF multimers were mainly associated with ADAMTS13. In conclusion, the threshold of shear rate must not exceed >6000 s^−1^ in order to maintain the optimal size of platelet aggregates to “plug off” the injury site and stop bleeding.

## 1 INTRODUCTION

Left ventricular assist device (LVAD) is a life‐saving tool for providing hemodynamic support in patients with advanced, refractory left ventricular heart failure, either for temporary support (eg, bridge to cardiac transplantation), or as permanent destination therapy.^1^, ^2^ Despite the enormous benefits of LVAD, bleeding remains the major cause of morbidity, mortality, and cost during LVAD support,^3^, ^4^ which cannot be attributed to anticoagulation alone.^5^, ^6^ The pathophysiologic mechanisms responsible for the bleeding diathesis in these patients have shown to be complex and poorly understood.

von Willebrand factor (vWF) acts as a bridging molecule at sites of vascular injury for normal platelet adhesion, as well as promoting platelet aggregation under a certain threshold of hydrodynamic forces,^7^ and is thus critical to hemostasis. However, supraphysiologic shear stress can cause pathologic vWF degradation during LVAD support.^8^, ^9^ High molecular weight (HMW) vWF multimers are the most hemostatically competent,^10^ and reduction in the HMW vWF multimers lead to impairment of vWF functional activity and platelet aggregation, which is associated with acquired von Willebrand Syndrome (aVWS).^11^, ^12^ During shear exposure, the unfolded vWF exposes its A2 domains, which are particularly sensitive to enzymatic cleavage by a disintegrin and metalloproteinase with a thrombospondin type 1 motif, member 13 (ADAMTS13).^9^, ^13^, ^14^ Subsequently, this causes excessive degradation of HMW vWF multimers. Such loss of HMW vWF caused by enzymatic cleavage of ADAMTS13 has been reported in in vitro LVAD evaluations.^9^, ^15^

The trigger threshold of shear‐induced vWF unfolding has been reported between 5000 and 5522 s^−1^ (Table 1).^16^, ^17^ This is approximately two to three times greater than the reported normal physiologic intravascular shear rate (<2000 s^−1^),^18^, ^19^, ^20^, ^21^ but lower than those generated by LVAD (ranging from 1429 to 171 428 s^−1^).^8^, ^22^, ^23^, ^24^, ^25^, ^26^, ^27^ While recent research established the relationship between shear rate and pathologic vWF degradation, precisely why bleeding complications only occur in some aVWS patients but not all remain unknown. An understanding of the formation size of platelet aggregates under different shear regimes might provide a new insight into how to solve this enigma.

**TABLE 1:** Shear rates and stresses within multiple scales of the normal body circulation, at sites of injury, and within ventricular assist devices. Shear stress is calculated with assuming viscosity of whole blood is 3.5 mPa·s

Shear condition	Blood vessel or device condition	Shear rate (s^−1^)	Shear stress* (Pa)	Reference
Normal physiological condition	In veins	15‐200	0.05‐0.7	18, 19, 20, 21
	Large arteries	286‐1142	1‐4	
	Micro arterioles	450‐2000	1.6‐7	
Shear‐induced unfolding trigger of vWF	Vascular injury site	5000‐5522	17.5‐19.3	16, 17
Supraphysiological condition	Axial‐flow LVAD	2571‐171 428	9‐600	8, 22, 23, 24, 25, 26, 27, 46, 47
	Centrifugal‐flow LVAD	1429‐65 714	5‐230	
	Toroidal‐flow LVAD	2286‐2857	8‐10	

Indeed, the study of shear stress and exposure time are frequently examined in LVAD, and the effects on the degradation of HMW vWF multimers are predominately reported.^9^, ^28^, ^29^, ^30^ However, no experimental investigation into the relationship between platelet aggregation size and shear rates has been reported. While our previous study observed smaller, more dispersed platelet aggregates at high shear rates as preliminary results,^31^ neither shear threshold nor real‐time visual investigation was established due to the limitation of previous blood shearing device. Hence, this study aimed to better understand how different mechanical shear stress and exposure time can alter the formation size of platelet aggregates in real‐time, and how it is linked to HMW vWF multimer cleavage by ADAMTS13 using a purpose‐built blood shearing device, which was incorporated with an inverted microscope. We hypothesized that the size of platelet aggregates is determined, in part, by shear history; specifically, when blood is exposed to shear beyond a critical threshold, platelet aggregate size and HMW vWF multimer size would be negatively impacted. The evaluation of platelet aggregate size provides an opportunity for novel approaches in detecting and monitoring bleeding due to pathological shear forces, particularly in those receiving LVAD therapy, extracorporeal membrane oxygenation therapy, and patients with severe aortic stenosis.^32^, ^33^, ^34^

## 2 MATERIALS AND METHODS

### 2.1 Blood collection

Fresh human whole blood (45 mL) from consenting healthy volunteers was collected (n = 5), and anticoagulated with citrate phosphate dextrose adenine (CPDA‐1) solution (Terumo Corporation, Tokyo, Japan) at a final concentration of 14% (v/v). Prior to blood collection, all volunteers were informed about the aims of the study in accordance with the Declaration of Helsinki. The experimental protocols were reviewed and approved by the Griffith University Human Research Ethics Committee (Protocol number 2018/730).

### 2.2 Preparation of fluorochrome‐labeled platelets and platelet‐poor plasma

Platelet‐rich plasma (PRP) was prepared by centrifuging whole blood for 10 minutes at 150 × *g* at room temperature. Ten milliters of PRP (500 000 platelets per μL) were stained with 50 μg of MitoTracker Red FM (Thermo Fisher Scientific, Waltham, MA, USA) fluorochrome at 37°C for 2 hours. Platelet‐specific staining protocols were previously described.^35^ After staining, fluorochrome‐labeled platelets were fixed with 1.37% formalin to maintain their membrane glycoprotein Ibα (GP Ibα) receptor function, which binds with vWF A1 domains during shear and form platelet aggregation. The remaining unstained PRP was further centrifuged for 5 minutes at 15 000 × *g* at room temperature to obtain platelet‐poor plasma (PPP) containing vWF.

### 2.3 Blood‐shearing device

**FIGURE 1:**
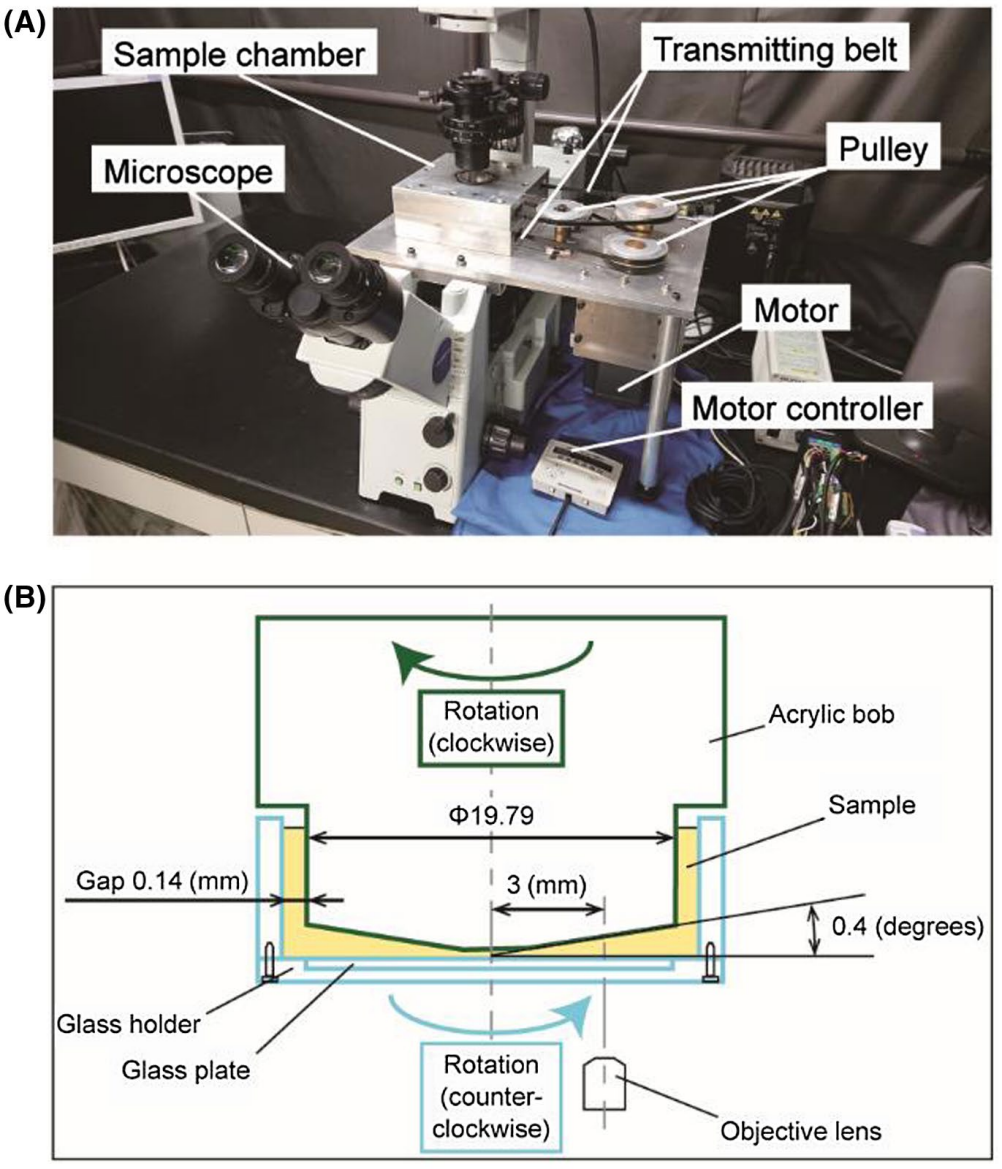
A, A custom‐built blood‐shearing device was designed to be incorporated into a microscope. When the motor rotates, two opposite rotational motions are generated and transmitted to the sample chamber using pulleys and transmitting belts. B, Cross‐sectional view of the sample chamber: an acrylic bob and a glass plate that rotates in the opposite direction to generate shear Couette flow. The formation of platelet aggregates in the sample chamber was visualized in real‐time through the ×40 objective lens of the microscope [Color figure can be viewed at wileyonlinelibrary.com]

Fluorochrome‐labeled platelets and PPP containing vWF with ristocetin were subjected to a wide range of shear‐controlled doses. Shear rates were generated at 0, 360, 1000, 3000, 6000, and 10 000 s^−1^ for 0, 5, 10, and 15 minutes using a custom‐built blood‐shearing device incorporated into an inverted fluorescence microscope (IX73, Olympus Corporation, Tokyo, Japan) for real‐time visualization of platelet‐vWF interactions shown in Figure 1A. The chosen shear rates in this study corresponded to shear stresses of 0, 0.5, 1.3, 3.8, 7.5, 12.5 Pa calculated from a viscosity of 1.25 mPa*·*s of PPP measured by a viscometer (DV2T, AMETEC Brookfield, Middleborough, MA, USA). Briefly, the shear rates were generated by the rotational speed of the motor (0, 10, 28, 83, 167, and 278 RPM), which is transmitted from the motor to the sample chamber using pulley and transmission belts. The sample chamber consists of two parts: an acrylic bob and a glass plate that rotate in the opposite direction to generate shear Couette flow (Reynold number within the sample chamber <100 even at the highest shear rate of 10 000 s^−1^)^36^ (Figure 1B). In the middle flow field at a radial distance of 3 mm from the axis is the observation target. The inner diameter of the acrylic bob is 19.79 mm and the gap size is 0.14 mm. The total loading sample volume is 200 μL. The rotating speed is adjustable from 10 to 278 RPM. This custom‐built blood‐shearing device was successfully used in previous studies.^37^, ^38^

### 2.4 Evaluation of platelet aggregation size using microscopy and image analysis

The platelets were diluted by imidazole buffered saline (IBS) and added into 200 μL of cell mixture (100 000 μL^−1^ of fluorochrome‐labeled platelets, 1 mg/dL of ristocetin, 10 μL of PPP, 130 μL of IBS). The formation of platelet aggregates under shear rates ranging from 360 to 10 000 s^−1^ was visualized in real‐time using a microscope with a ×40 magnification objective lens (LUCPLFLN 40X, Olympus Corporation) through acquired images of 162.5 nm/pixel resolution. MATLAB software (version R2018a, Mathworks, Natick, MA, USA) was used to remove noise on images through binarization of image data and to transform the number of pixels of aggregation area to the actual size of platelet aggregates (μm^2^). The aggregate size is measured as a two‐dimensional area (surface area). In addition, the vWF function was assessed using the vWF ristocetin cofactor assay.^35^

### 2.5 Gel electrophoresis and immunoblotting for high molecular weight vWF multimers

Electrophoresis was performed as previously described.^39^, ^40^ Briefly, PPP samples were run through a high gelling temperature SeaKem agarose gel (Lonza, Basel, Switzerland) and transferred to an Immobilon‐P transfer membrane (Merck KGaA, Darmstadt, Germany). Membranes were blocked using 5% skim milk in Tris Buffered Saline with Tween (Sigma Aldrich, St. Louis, MO, USA) and probed with horseradish peroxidase‐conjugated polyclonal rabbit anti‐human vWF (1:1000) (DAKO, Glostrup, Denmark). Membranes were washed and developed with enhanced chemiluminescence (Clarity ECL Western Blotting Substrate, Bio‐Rad Laboratories, Inc, Hercules, CA, USA), and vWF multimer band density visualized with an ImageQuant LAS 4000 (GE Healthcare, Chicago, IL, USA). The densities of HMW vWF multimers were quantified using standard software (Image J, version 1.8.0_112, National Institute of Health, Bethesda, MD, USA). The HMW vWF multimer density of each condition sample was normalized to the static (0 s^−1^) samples as a baseline and expressed as mean fold change ± standard deviation.

### 2.6 Investigation of the influence mechanism of platelet aggregation size and degradation of HMW vWF multimers

To investigate the influence of mechanical shear and enzymatic cleavage mechanism on platelet aggregation size, 10 mM of ethylenediaminetetraacetic acid (EDTA) (Thermo Fisher Scientific, Waltham, MA, USA) was used as an inhibitor for ADAMTS13 to prevent enzymatic cleavage of vWF.^29^ The size of platelet aggregates was then obtained via microscopy and image analysis under a low and high shear rate of 360 and 10 000 s^−1^ for 15 minutes with or without the presence of EDTA using the same method mentioned above. Shear rates of 0 and 360 s^−1^ were used as negative and positive controls. In addition, we also use the vWF multimer analysis to double confirm this influence.

### 2.7 Statistical analysis

Equality of variances for two groups was assessed for each condition by *F* test. Additionally, if variances were considered as equal, one‐way ANOVA tests were used to analyze the mean difference between two groups of specific exposure time. All statistical analysis was performed using JMP Pro (version 14 SAS Institute, Cary, NC, USA). Data are described as mean ± standard deviation (SD), and *P* values <.05 are considered statistically significant.

## 3 RESULTS

### 3.1 Visual investigation of formation size of platelet aggregates measured by microscopy

Captured images of platelet aggregates at different shear rates (0‐10 000 s^−1^) and exposure time (0‐15 minutes) are provided in Table 2. Without shear, there was no formation of platelet aggregates (<50 µm^2^). Large platelet aggregates were formed (>200 µm^2^) from 360 to 3000 s^−1^ of shear rate between 5 and 15 minutes. The average formation size of platelet aggregates was significantly smaller (<100 µm^2^) when the shear rate was higher than 6000 s^−1^ compared to the results from 360 to 3000 s^−1^ (*P* < .05).

**TABLE 2:**
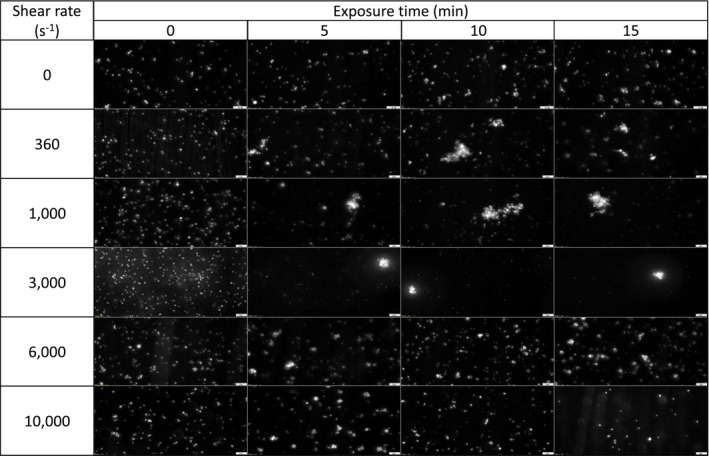
Captured images (× 40 objective) of platelet aggregates by microscopy at different shear rates (0‐10,000 s^−1^) and exposure time (0‐15 min). Platelet aggregates were not observed in no‐shear environments (<50 μm^2^). During low shear conditions ranging from 360 to 3,000 s^−1^, large platelet aggregates were observed (> 200 μm^2^); whereas smaller platelet aggregates (< 100 μm^2^) were observed in high shear conditions from 6,000 to 10,000 s^−1^

**FIGURE 2:**
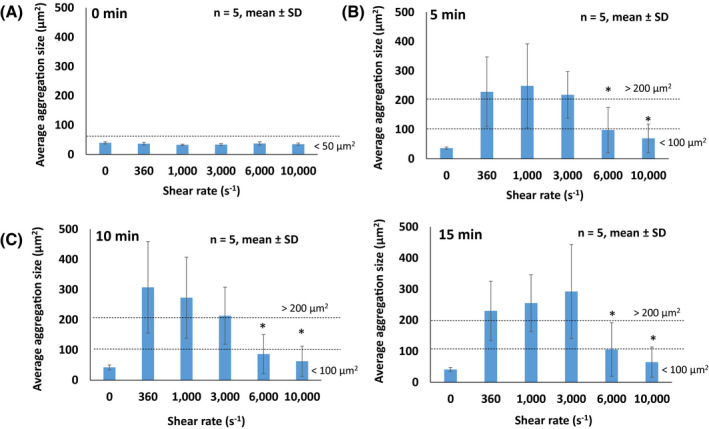
The effect of shear rate on average platelet aggregation size measured through microscopy (A‐D). Average platelet aggregation size formed under different shear rates of 0, 360, 1000, 3000, 6000, 10 000 s^−1^ at different exposure times. Results are described as mean ± standard deviation (SD), n = 5, *P < .05 [Color figure can be viewed at wileyonlinelibrary.com]

**FIGURE 3:**
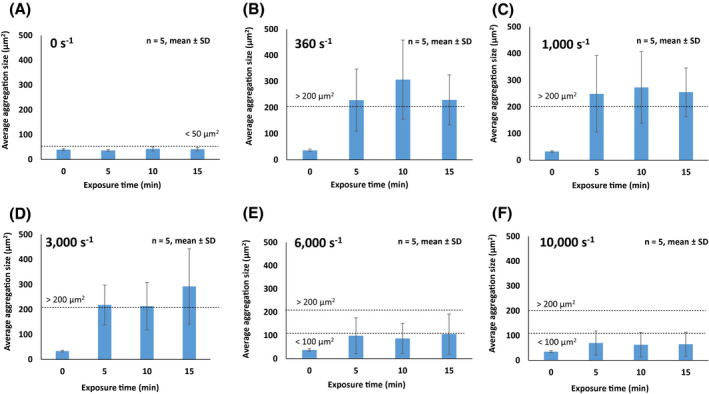
The effect of exposure time on average platelet aggregation size measured through microscopy (A‐D). Average platelet aggregation size formed under different shear rates at different exposure times of 0, 5, 10, and 15 minutes. Results are described as mean ± standard deviation (SD), n = 5, *P < .05 [Color figure can be viewed at wileyonlinelibrary.com]

In the absence of shear rate, the platelet aggregation size was minute with an average size smaller than 50 µm^2^ (Figure 2A). While platelet aggregates larger than 200 µm^2^ were observed when sheared between 360 and 3000 s^−1^, the average aggregate size of platelets sheared at 6000 and 10 000 s^−1^ were significantly reduced (*P* < .05), respectively (Figure 2B‐D). When platelets were exposed to higher shear rates at 6000 and 10 000 s^−1^ for 15 minutes, the average platelet aggregate size decreased to 100.1 ± 8.2 and 65.7 ± 4.0 μm^2^, respectively. However, the results in Figure 3 show that the effect of exposure time has minimal influence on the aggregate size compared to the shear rate. In Figure S1, the result of flow cytometric investigation of the vWF function shows that there were no significant differences in normalized platelet aggregation between the different shear conditions.

### 3.2 Quantification of the loss of HMW vWF multimers using densitometry

**FIGURE 4:**
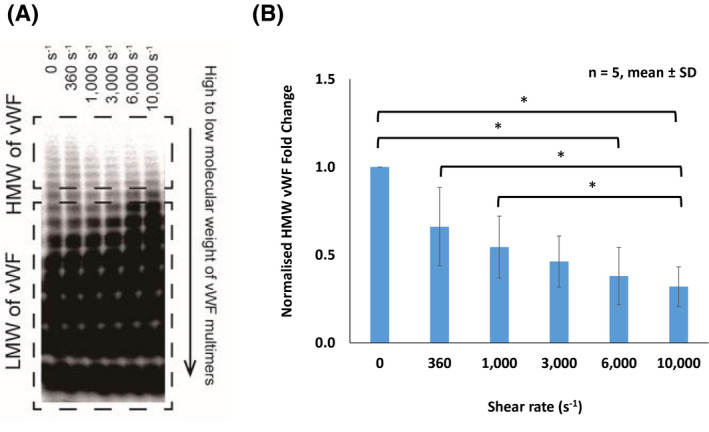
A, von Willebrand factor (vWF) multimer analysis demonstrates high molecular weight (HMW) of vWF multimers degraded as the increasing a shear range of 0‐10 000 s^−1^ for an exposure time of 15 minutes. HMW of vWF multimers degraded (top dotted box) and low molecular weight (LMW) of vWF multimers accumulated as shear increased (bottom dotted box). B, High molecular weight von Willebrand factor (HMW vWF) density obtained from immunoblotting at shear range 0‐10 000 s^−1^ quantified using densitometry. HMV vWF density (360‐10 000 s^−1^) were normalized to the static result (0 s^−1^) as a baseline and described as mean ± standard deviation (SD), n = 5, *P < .05 [Color figure can be viewed at wileyonlinelibrary.com]

vWF multimer analysis revealed that HMW vWF multimers (top dotted box) were cleaved into low‐molecular‐weight (LMW) vWF multimers (bottom dotted box) as the shear rate increased (Figure 4A). The result of densitometry showed that HMW vWF multimer degradation was evident when mechanical shear increased (Figure 4B). There was significant loss of HMW vWF under shear rates of 6000 and 10 000 s^−1^ for 15 minutes by 62% and 68%, respectively, compared to those without shear (0 s^−1^) (*P* < .05). Furthermore, at a maximum shear rate of 10 000 s^−1^, the density in HMW vWF shows it to be significantly lower than those sheared at 360 and 1000 s^−1^ (*P* < .05).

### 3.3 The effect of ADAMTS13 enzymatic cleavage on platelet aggregation size and degradation of HMW vWF multimers

**FIGURE 5:**
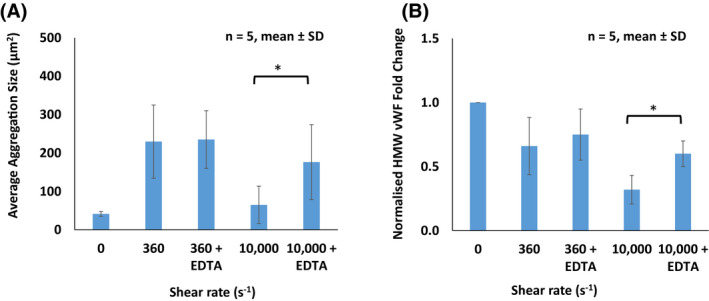
A, Average platelet aggregation size formed following shearing at 360 and 10 000 s^−1^ for an exposure time of 15 minutes with and without ethylenediaminetetraacetic acid (EDTA) (ADAMTS13 inhibitor) to determine the contribution between ADAMTS13 and the induced shear force on the reduced platelet aggregation size. Results are described as mean ± standard deviation (SD), n = 5, *P < .05. B, von Willebrand factor (vWF) multimer analysis demonstrates degradation of high molecular weight von Willebrand factor (HMW vWF) multimers following shearing at 360 and 10 000 s^−1^ for an exposure time of 15 minutes with and without EDTA (ADAMTS13 inhibitor) to determine the contribution between ADAMTS13 and the induced shear force on the degradation of HMW vWF multimers. Results are described as mean ± standard deviation (SD), n = 5, *P < .05 [Color figure can be viewed at wileyonlinelibrary.com]

In Figure 5A, the average platelet aggregation size was significantly lower in the absence of EDTA (64.9 μm^2^) compared to those with EDTA (176.3 μm^2^) following exposure to a high shear rate of 10 000 s^−1^ for 15 minutes (*P* < .05). Whereas, no significant difference was found in the average platelet aggregation size following exposure to a low shear rate of 360 s^−1^ for 15 minutes with (235 μm^2^) and without EDTA (229.7 μm^2^). In Figure 5B, the normalized HMW vWF fold change was significantly lower in the absence of EDTA (0.32) compared to those with EDTA (0.60) following exposure to a high shear of 10 000 and at 0 s^−1^ (*P* < .05). No significant differences were found in the normalized HMW vWF fold change following exposure to a low shear rate of 360 s^−1^ for 15 minutes with (0.68) and without EDTA (0.66).

## 4 DISCUSSION

During vascular injury, the shear‐induced unfolding of vWF has a central role in primary hemostasis where it mediates platelet adhesion to damaged vascular subendothelium and subsequently platelet aggregation.^7^ This process has long been recognized as critical for hemostatic plug formation and to stop bleeding.^41^ Although many studies reported the effect of shear rate and exposure time on the degradation of HMW vWF multimers, the effect of shear rate and exposure time on the formation size of platelet aggregates has never been reported experimentally. Our results demonstrate, for the first time that a critical shear threshold exists that determines platelet aggregate formation size, which extends prior knowledge on the effects of shear on HMW vWF degradation. This shear‐dependent platelet aggregation size could be one of the determinants that underpin bleeding events in LVAD patients.

Our result showed that above shear rate of 6000 s^−1^, the average size of observed platelet aggregates (<100 µm^2^) was significantly smaller than the platelet aggregates formed (>200 µm^2^) under shear rate of 3000 s^−1^. Smaller platelet aggregates were observed in patients after cardiopulmonary bypass.^42^ When vWF is exposed to shear, the vWF uncoils and its string length can stretch either >200 µm (uncleaved ultra‐large vWF) or <100 µm (cleaved vWF fragments) which are consistent with our reported size of platelet aggregates.^43^ With longer vWF string length stretches, as it exposes greater access to binding sites (GP Ibα), more platelets can adhere and hence larger platelet aggregates can form. Therefore, the formation of platelet aggregation size under different shear conditions could be an important parameter to predict and stop bleeding during vascular injury.^41^ In addition, two studies reported similar thresholds of shear‐induced hydrodynamic activation of vWF.^16^, ^17^ Hence, we believe that the threshold of shear‐induced unfolding trigger of vWF and lead to reduction in the size of platelet aggregates is between 3000 and 6000 s^−1^.

It was previously demonstrated that the losses of HMW vWF under shear rates of 4000 and 8000 s^−1^ for 360 minutes were approximately 55% and 80%, respectively.^31^ Our vWF multimer analysis result showed that the losses of HMW vWF under shear rates of 6000 and 10 000 s^−1^ for 15 minutes were 62% and 68%, respectively. These results indicate that the shear rate and exposure time are important contributors in the degradation of HMW vWF multimers. Moreover, while the multimers of HMW vWF monotonically deteriorated as shear rate increased, the aggregate size was clearly reduced when the shear rate was over 6000 s^−1^. These results infer that platelet‐vWF binding may be prevented in shears >3000 s^−1^, possibly due to strong hydrodynamic forces.^44^, ^45^ Furthermore, EDTA was used to inhibit ADAMTS13, which facilitated confirmation that decreased platelet aggregate size in higher shears was, indeed, associated with the degradation of HMW vWF multimers mediated by ADAMTS13 cleavage.^9^

### 4.1 Limitations

Within this study, three limitations were identified. First, the current shear device is limited to generate a maximum shear rate of 10 000 s^−1^, where the entire range of shear rates (1429 to 171 428 s^−1^) reported in the current‐generation axial and centrifugal‐flow LVADs (Table 1) was unable to be assessed. However, shear rates investigated in this study represent more than 95% relative volume distribution of scalar shear rates ranging from 1429 to 14 286 s^−1^.^25^, ^26^, ^27^ Of note, the new design of next‐generation LVADs such as toroidal‐flow LVAD has focused on providing full hemodynamic support with lower shear rate of approximately 2857 s^−1^ to reduce blood trauma.^46^, ^47^ Second, our current blood‐shearing experiments were performed at room temperature instead of body temperature (37°C) and aggregate size was assessed from two‐dimensional images rather than as three‐dimensional volumes. Third, the current work was focused on understanding the mechanobiology of vWF multimers interaction with platelets under shear and how that influences the formation sizes of platelet aggregates. However, platelet function was not assessed in this study which also contributes and influences the formation sizes of platelet aggregates. Therefore, the effect of shear rate and exposure time on the platelet function should be considered in future research. We will also investigate the effect of pulsatility on the formation size of platelet aggregates, platelet function, and vWF degradation because pulsatility plays an important role in the phasicity of intravascular shear stress and may have important relationships with vWF degradation and bleeding.^48^, ^49^

## 5 CONCLUSIONS

Our results suggested that the maximum threshold of shear rate was between 3000 and 6000 s^−1^ to maintain the normal size of platelet aggregates (>200 µm^2^). There is a correlation between the size formation of platelet aggregates and HMW vWF multimer loss following exposure to shear. In addition, the reduced platelet aggregation size and HMW vWF multimers were associated with shear‐induced ADAMTS13 cleavage. In conclusion, the evaluation of platelet aggregate size provides an opportunity for novel approaches in detecting and monitoring bleeding due to pathological shear forces, particularly in those receiving LVAD therapy, extracorporeal membrane oxygenation therapy, and patients with severe aortic stenosis.

## CONFLICT OF INTEREST

The authors declare that they have no conflicts of interest with the contents of this article.

## AUTHOR CONTRIBUTIONS

*Concept/design, analysis/data interpretation, secured funding, drafting/critical revision/approval of the article:* Chan

*Analysis/data interpretation, statistics, drafting/approval of the article:* Inoue

*Critical revision/approval of the article:* Ki

*Analysis/data interpretation, approval of the article:* Murashige

*Secured funding, approval of the article:* Fraser

*Concept/design, critical revision/approval of the article:* Simmonds, Tansley, Watanabe

## Supporting information

Fig S1
