# Supplementary figures and images for: Shear‐dependent platelet aggregation size
Source: Artif Organs. 2020 Aug 15;44(12):1286–95. doi: 10.1111/aor.13783 (PMC7818454; doi:10.1111/aor.13783)

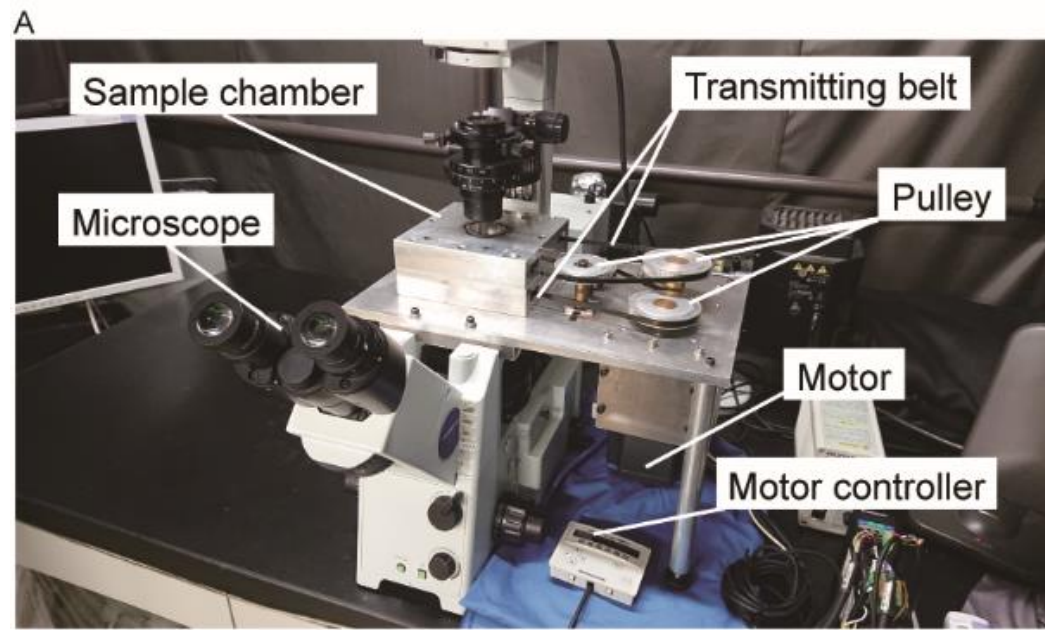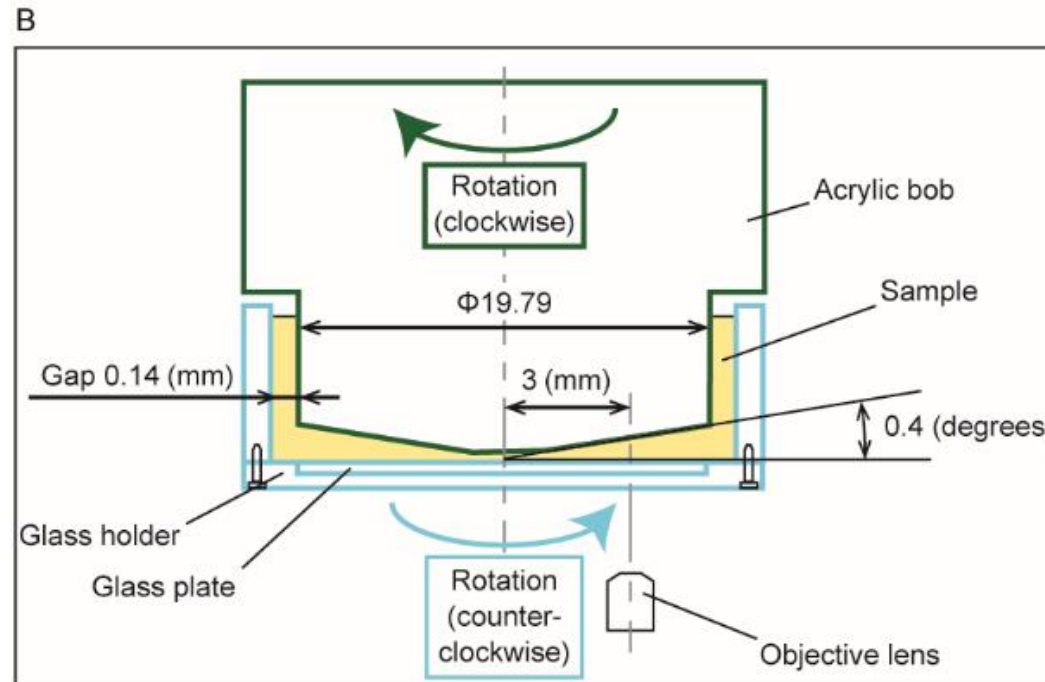

**Figure 1.**

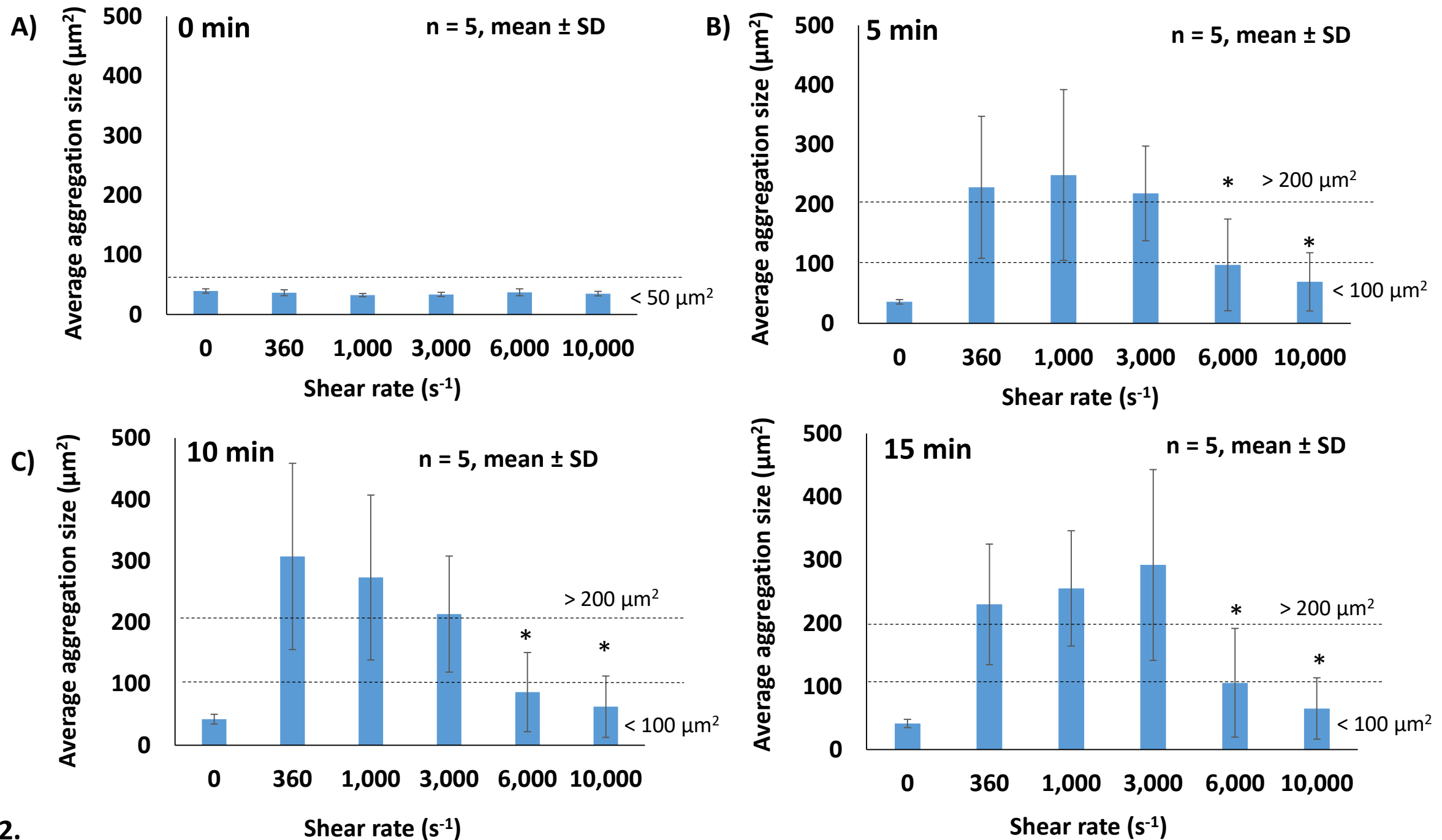

Figure 2.

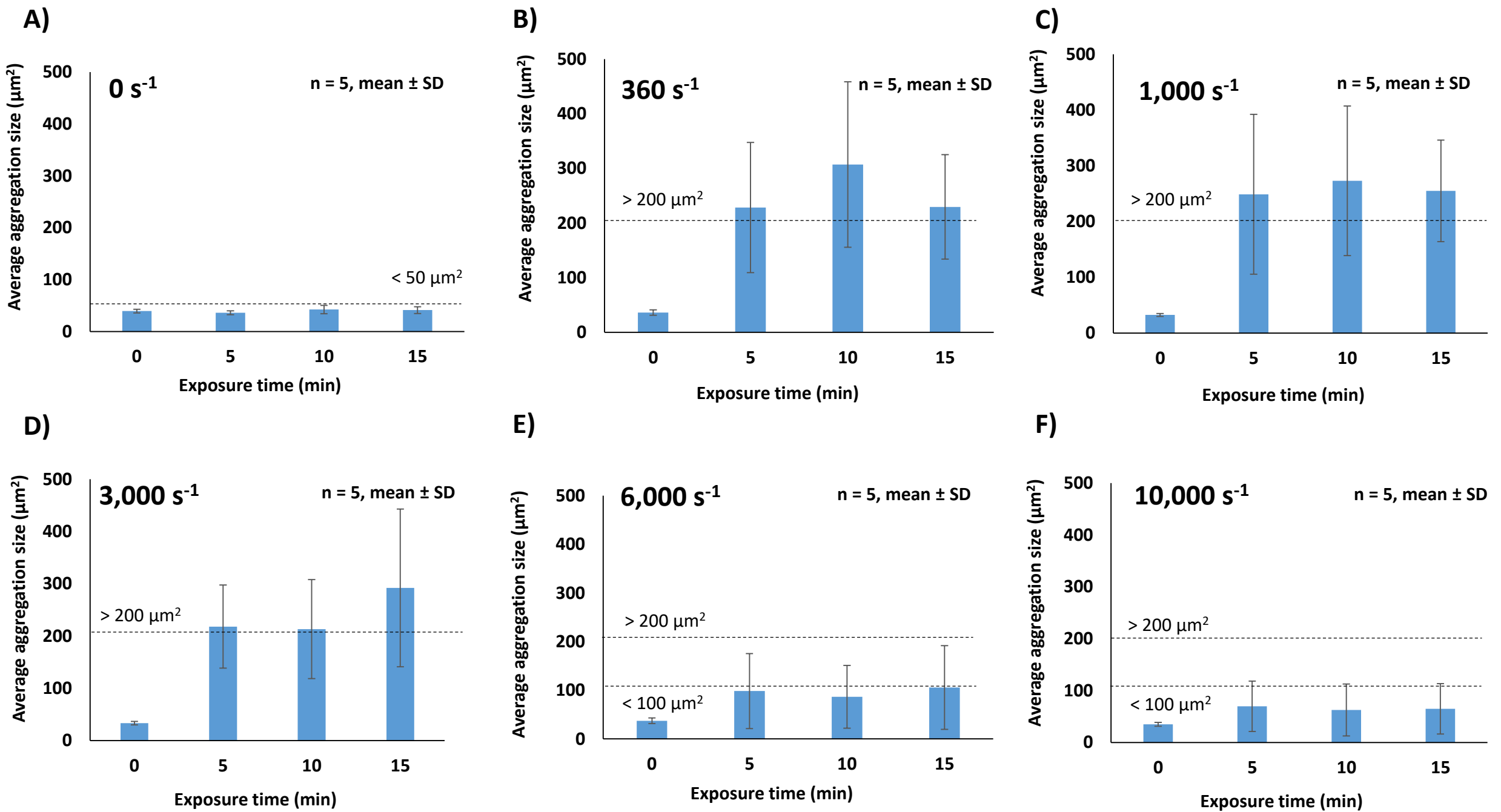

Figure 3.

A)

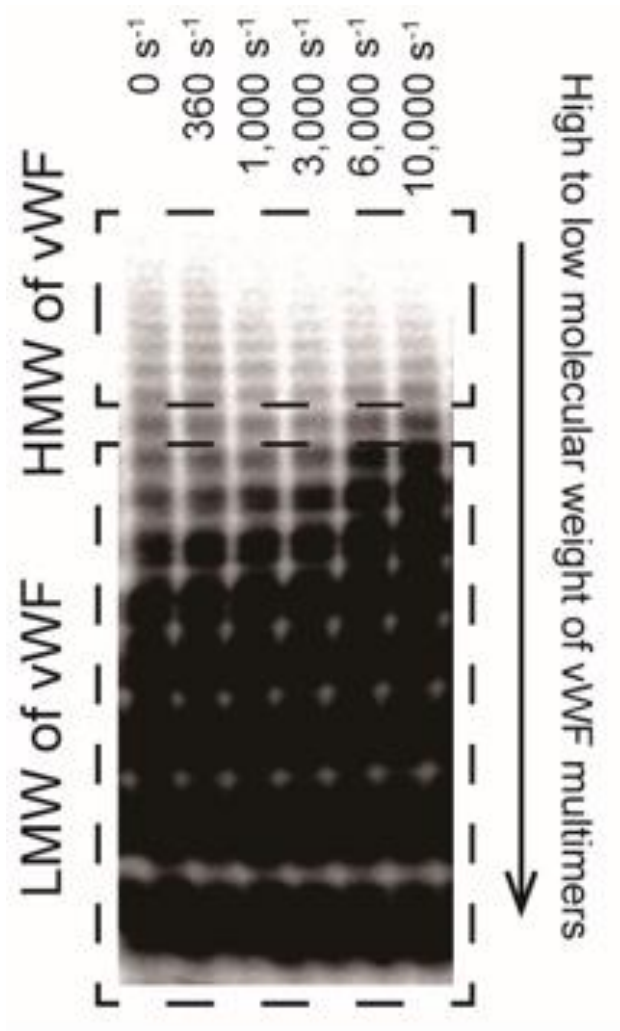

B)

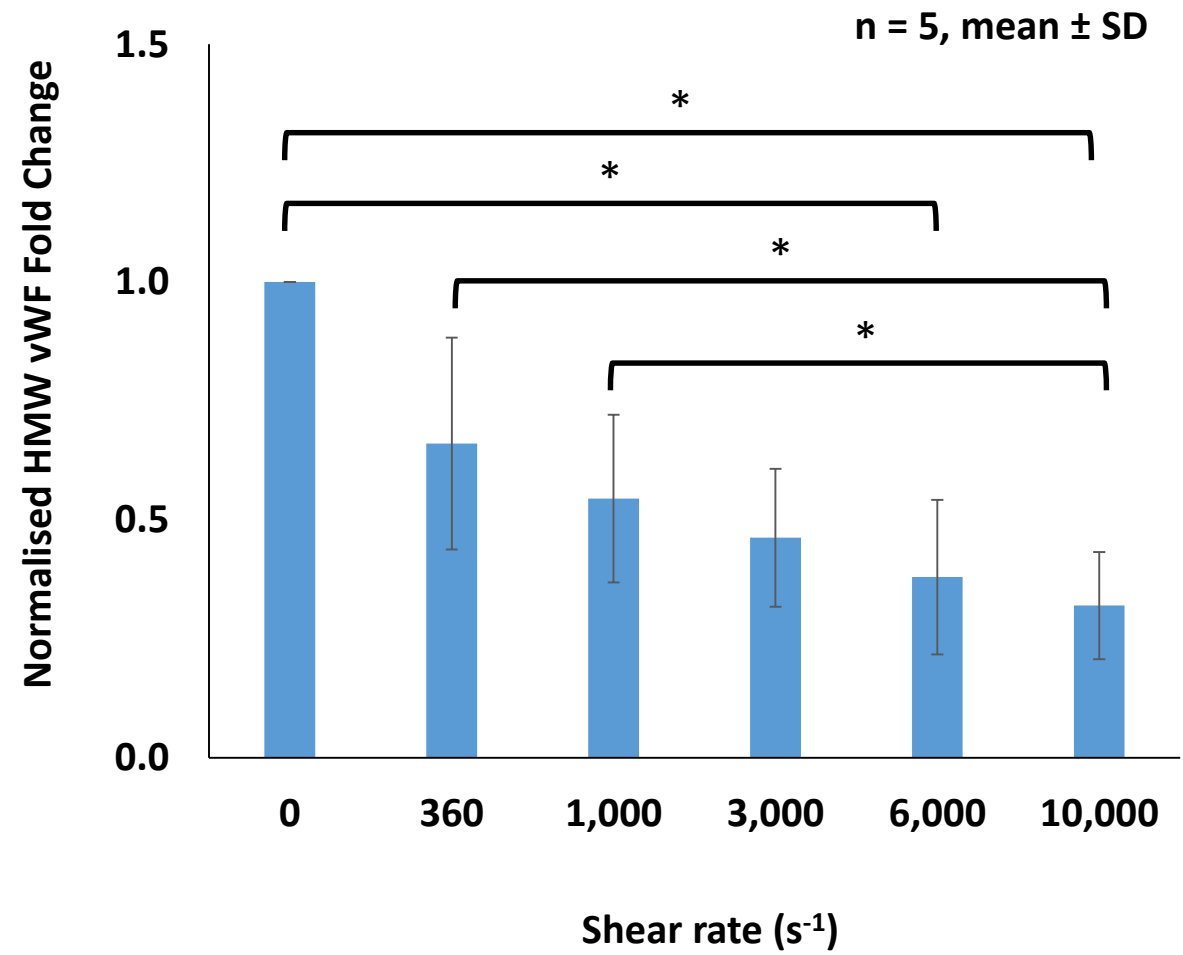

Figure 4.

A)

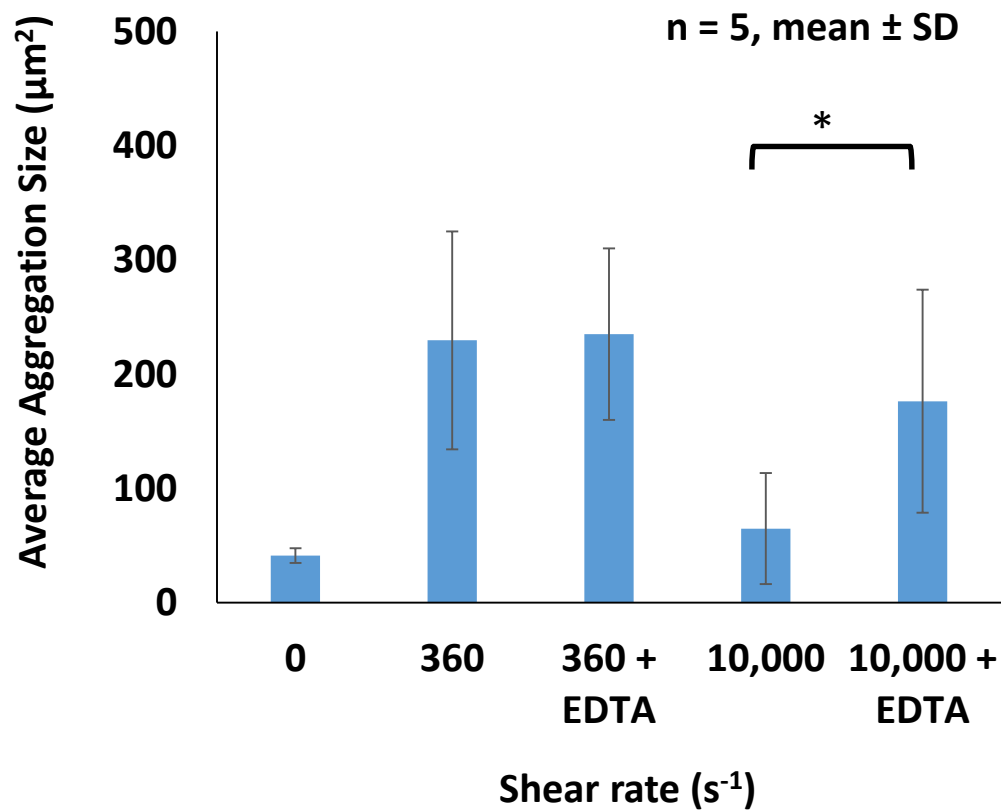

B)

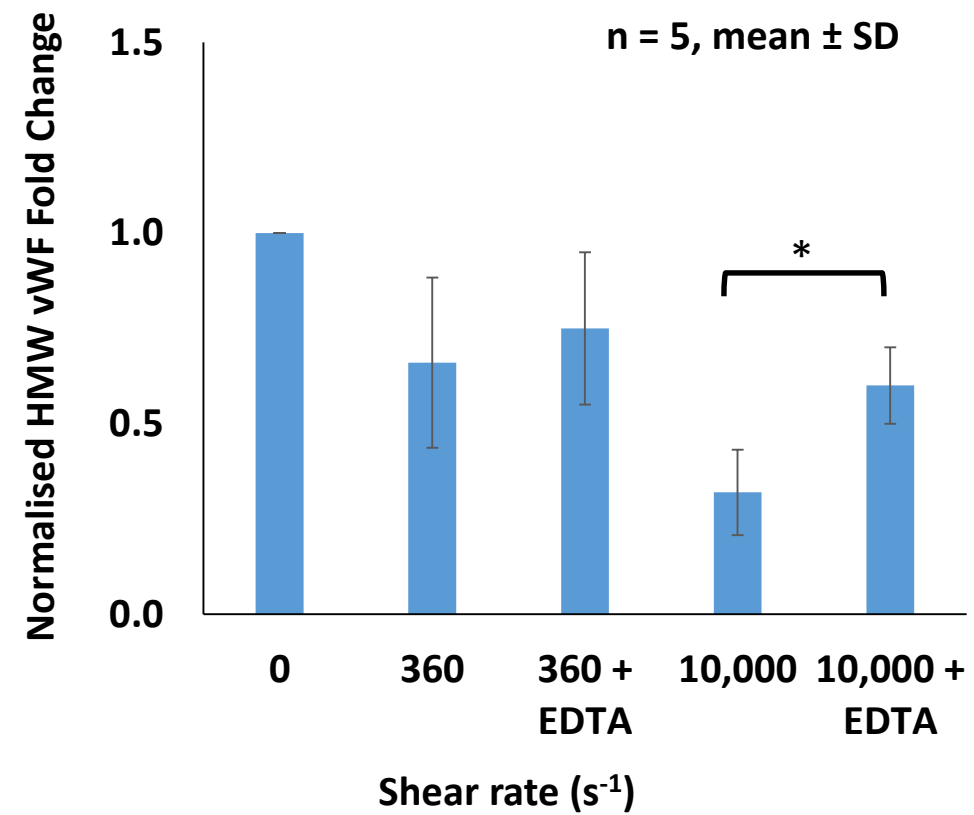

Figure 5.

A

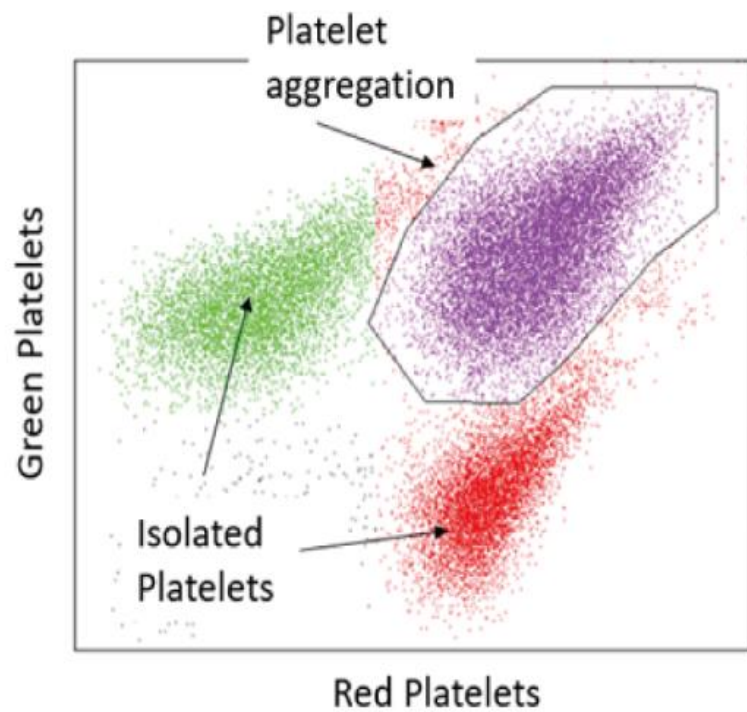

B

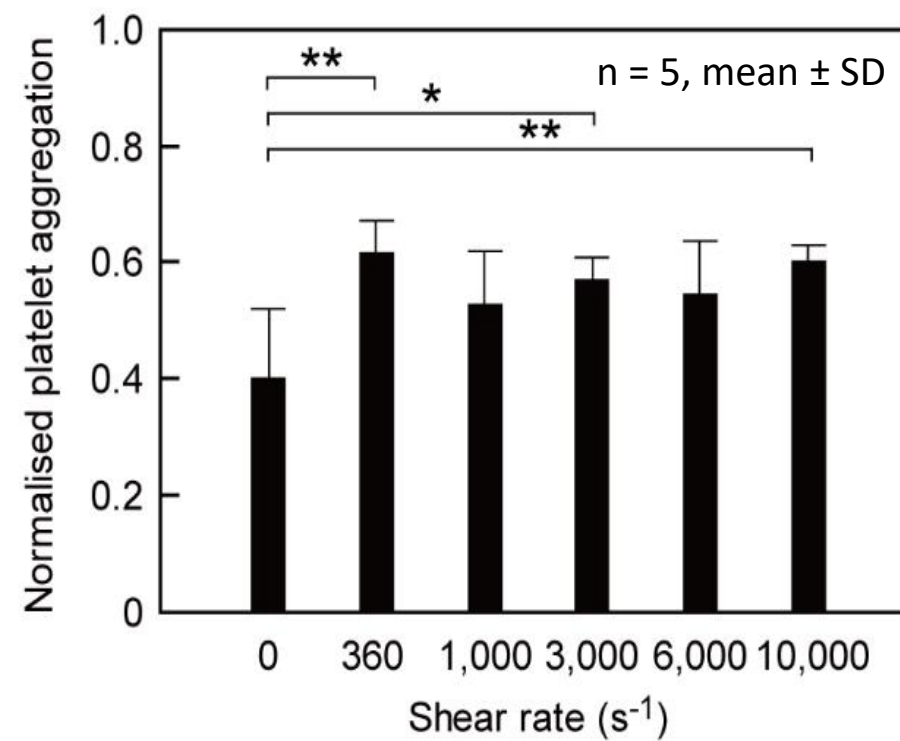

Supplemental Figure 1.

Supplement: Supplementary file 1 — Fig S1 [file AOR-44-1286-s001.pdf]
